# *ZmEREB180* modulates waterlogging tolerance in maize by regulating root development and antioxidant gene expression

**DOI:** 10.1111/pbi.70030

**Published:** 2025-03-09

**Authors:** Huanhuan Qi, Jing Wang, Xin Wang, Kun Liang, Meicheng Ke, Xueqing Zheng, Wenbin Tang, Ziyun Chen, Yinggen Ke, Pingfang Yang, Fazhan Qiu, Feng Yu

**Affiliations:** ^1^ State Key Laboratory of Biocatalysis and Enzyme Engineering, School of Life Science Hubei University Wuhan China; ^2^ School of Life Science and Technology Wuhan Polytechnic University Wuhan China; ^3^ National Key Laboratory of Crop Genetic Improvement, Hubei Hongshan Laboratory Huazhong Agricultural University Wuhan China

**Keywords:** maize, *ZmEREB180*, root development, antioxidant genes

With climate change increasing the frequency of extreme weather events, waterlogging has become a significant threat to agricultural production, especially in maize‐growing regions. Waterlogging induces hypoxic conditions in the root zone, limiting maize growth and yield (Liang *et al*., 2020; Pedersen *et al*., 2017). Plants have evolved adaptive mechanisms, such as adventitious root (AR) formation and enhanced antioxidant activity, to cope with waterlogging stress (Pedersen *et al*., 2021; Yamauchi *et al*., 2018). However, the regulatory mechanisms in maize remain poorly understood.

Group VII ethylene response factor proteins (ERFVIIs) are key regulators of waterlogging tolerance in model plants (Hartman *et al*., 2021). Our previous work showed that *ZmEREB180*, a maize ERFVII, promotes waterlogging tolerance by enhancing AR formation and modulating antioxidant levels (Yu *et al*., 2019). In this study, we cloned the full‐length coding sequence of *ZmEREB180* and inserted it into the pM999 vector. The recombinants and empty vector were transiently expressed in isolated B73 leaf protoplasts, followed by a transient and simplified cleavage under targets and tag‐mentation (tsCUT&Tag) assay (Liang *et al*., 2024). A total of 4720 confident peaks corresponding to 3335 genes were identified (Table S1). Notably, 70.15% of these peaks were located in promoter regions, with 68.67% found in promoters less than 1 kb upstream (Figure 1a). The highest enrichment was observed at the transcription start site (Figure 1b). Motif analysis revealed the GCC‐box (GCCGCC) as the highest scoring motif (E‐value = 5.7 × 10^−10^). Compared with RNA‐Seq data (Yu *et al*., 2019) identified 421 genes that were differentially expressed in the *ZmEREB180* overexpression lines, under waterlogged conditions, and were directly bound by ZmEREB180 (Figure 1c; Table S2). We focused on genes involved in root development and antioxidant pathways. Lateral organ boundaries domain (LBD) proteins play pivotal roles in organ development. Two LBD genes, *ZmLBD5* and *ZmLBD38* (Table S2), were up‐regulated in an overexpression line and under waterlogging conditions, in which *ZmLBD5* has been shown to promote AR formation (Feng *et al*., 2022). Four antioxidant genes, including two glutathione‐S‐transferases (GST, *ZmGST8* and *ZmGST31*) and two peroxidases (POD, *ZmPOD12* and *ZmPOD55*), exhibited similar expression profiles (Table S2). The tsCUT&Tag data revealed significant peaks in the promoter of these genes (Figure 1d). Additionally, GCC‐box motifs were located in these regions, suggesting direct regulation by ZmEREB180 under waterlogging conditions.

**Figure 1:**
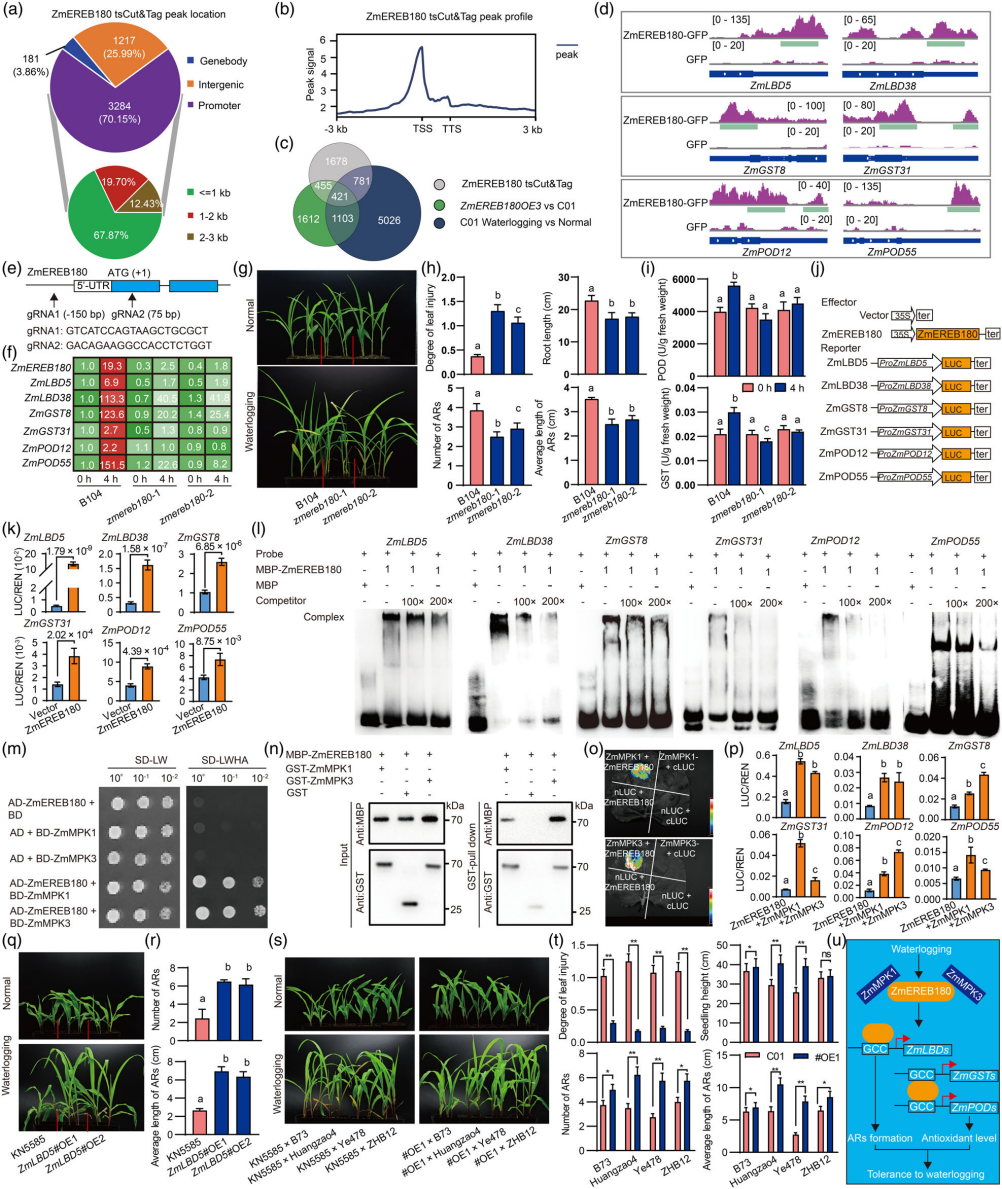
The regulatory networks of *ZmEREB180* involved in maize waterlogging tolerance. (a) Distribution of identified peaks from ZmEREB180 tsCut&Tag. Two independent experiments were conducted and the overlapping genes were used for further analysis. (b) Peak profile flanking the 3‐kb gene body. TSS, transcript start site; TTS, transcription termination site. (c) Overlap of genes identified by ZmEREB180 tsCut&Tag and differentially expressed genes in *ZmEREB180* overexpression lines and in waterlogging stress. (d) Distribution of ZmEREB180 binding sites in six target genes. (e) Design for gene editing of *ZmEREB180*. (f) Expression levels of *ZmEREB180* and its target genes in *zmereb180* mutants and B104 roots under normal (0 h) and waterlogging conditions (4 h). The gene expression values represent the mean derived from three independent biological replicates. (g) Phenotypes of *zmereb180* mutants and B104 under normal conditions and after 6 days of waterlogging, along with leaf and root characterization (h) and corresponding physiological responses, including POD and GST activity (i). (j, k) Luciferase activity of ZmEREB180 effector and its targets in maize protoplasts. (l) Electrophoretic mobility shift assay demonstrating the binding of ZmEREB180 to the promoters of its target genes. (m) Yeast two‐hybrid assay to validate the interaction between *ZmEREB180* and ZmMPK1/ZmMPK3. (n) In vitro pull‐down assay confirming the interaction between ZmEREB180 and ZmMPK1/ZmMPK3. (o) Split luciferase assay to further validate the interaction between ZmEREB180 and ZmMPK1/ZmMPK3. (p) Luciferase activity of ZmEREB180 effector combined with ZmMPK1, ZmMPK3, and their target genes in maize protoplasts. (q) Phenotypes of *ZmLBD5* overexpression lines and wild‐type KN5585 under normal and 6 days of waterlogging conditions, along with root characterization (r). (s) Phenotypes of F1 hybrids derived from KN5585 and ZmLBD5#OE1 crossed with four inbred lines, under normal and 10 days of waterlogging conditions, along with leaf and root characterization (t). (u) Regulatory networks mediated by ZmEREB180.

Two CRISPR/Cas9‐generated mutants, *zmereb180*‐1 and *zmereb180*‐2, in maize line B104 were analysed (Figures 1e and S1). The expression level of *ZmLBD5*, *ZmLBD38* and the antioxidant genes was downregulated in both two mutants under waterlogging (Figure 1f; Table S3), indicating ZmEREB180 regulates these genes. No differences were observed between B104, *zmereb180*‐1 and *zmereb180*‐2 at the second leaf stage, but significant phenotypic differences were evident after 6 days of waterlogging treatment (Figures 1g and S2). Both mutants showed greater leaf injury, reduced root length, AR number, and AR length compared to B104 (Figure 1h). Increased POD and GST activity in B104 roots under waterlogging was diminished in the mutants (Figure 1i), supporting the role of *ZmEREB180* in waterlogging tolerance.

To confirm the binding of ZmEREB180 to these targets, we performed dual luciferase reporter assays using 1.5‐kb promoter fragments of *ZmLBD5*, *ZmLBD38*, *ZmGST8*, *ZmGST31*, *ZmPOD12* and *ZmPOD55* (Figure 1j). Co‐transfection with ZmEREB180 in maize leaf protoplasts significantly enhanced the expression of all six genes, especially *ZmLBD5*, *ZmLBD38* and *ZmGST8* (Figures 1k and S3). Electrophoretic mobility shift assays (EMSAs) confirmed that ZmEREB180 binds directly to the GCC motifs in the promoters of these genes (Figures 1l and S4).

A time‐course transcriptome analysis of waterlogged roots in B73 seedlings revealed a significant enrichment of mitogen‐activated protein kinase (MPK) signalling under stress (Yu *et al*., 2020). Using a yeast two‐hybrid assay, we identified two MPKs, ZmMPK1 and ZmMPK3, that interacted with ZmEREB180 (Figure 1m). GST pull‐down assays confirmed that ZmEREB180 interacts with ZmMPK1 and ZmMPK3 in vitro (Figure 1n). A split luciferase assay further validated these interactions in plata (Figure 1o). Co‐transfection of ZmMPK1 or ZmMPK3 with ZmEREB180 in maize leaf protoplasts significantly enhanced the activation of *ZmLBD5*, *ZmLBD38*, *ZmGST8*, *ZmGST31*, *ZmPOD12* and *ZmPOD55* promoters (Figure 1p), suggesting that ZmMPK1 and ZmMPK3 enhance ZmEREB180‐mediated transcriptional activation.

To access the functional role of ZmEREB180 target genes under waterlogging, we subjected *ZmLBD5* overexpression lines to waterlogging treatment. Overexpression lines, *ZmLBD5*#OE1 and *ZmLBD5*#OE2, showed significantly enhanced AR formation, seedling growth, and waterlogging tolerance after 6 days of stress, compared with wild‐type KN5585 (Figures 1q, r, S5 and S6). F1 hybrids from a cross of *ZmLBD5*#OE1 and four maize inbred lines (B73, Huangzao4, Ye478 and ZHB12) also displayed improved waterlogging tolerance after 10 days of treatment (Figure 1s, t), with reduced leaf injury and enhanced root and seedling growth.

Our findings highlight the critical role of *ZmEREB180* in regulating maize tolerance to waterlogging stress (Figure 1u). We demonstrate that *ZmEREB180* directly interacts with key genes such as *ZmLBD5*, *ZmLBD38*, *ZmGST8*, *ZmGST31*, *ZmPOD12* and *ZmPOD55*, positively modulating their expression under waterlogging. We also show that interactions with ZmMPK1 and ZmMPK3 enhance the activation of downstream target genes. Overexpression of *ZmLBD5* improves AR formation and waterlogging tolerance across different genetic backgrounds, making it a promising target for developing maize varieties with improved resilience to waterlogging, major abiotic stress affecting global maize production.

## Author contributions

FY and FQ designed the project. HQ and XZ performed data analysis. JW, XW and ZC conducted the experiments. KL, MK and WT participated in some experiments. HQ and FY wrote the original manuscript. YK and PY contributed to the interpretation of data. FY and FQ revised the manuscript.

## Conflict of interest

No conflict of interests to declare.

## Supporting information


**Figure S1** The sequence variations in *zmereb180* mutants.

**Figure S2** The phenotypes of leaves (A) and roots (B) in B104 and *zmereb180* mutants under normal and waterlogged conditions. The red arrows indicate the regions where adventitious roots are growing.

**Figure S3** The expression level of *ZmEREB180* in protoplast using qRT‐PCR. Five independent replicates were performed for each effector.

**Figure S4** The probe sequence directs the location of the GCC‐box used for EMSA analysis.

**Figure S5** The phenotypes of roots in KN5585, *ZmLBD5*#OE1 and *ZmLBD*#OE2 under normal and waterlogged conditions. The red arrows indicate the regions where adventitious roots are growing.

**Figure S6** The height and fresh weight of seedlings in *ZmLBD5* overexpression lines.

**Table S1** The peaks identified in ZmEREB180 tsCut&Tag.

**Table S2** The overlapped genes in ZmEREB180 binding, ZmEREB180 overexpression line and under waterlogging stress.

**Table S3** The primers for the qRT‐PCR used in the present study.

## Data Availability

The data that support the findings of this study are available in the supplementary material of this article.
