# Supplementary material for: ZmEREB180 modulates waterlogging tolerance in maize by regulating root development and antioxidant gene expression
Source: Plant Biotechnol J. 2025 Mar 9;23(6):2062–4. doi: 10.1111/pbi.70030 (PMC12120909; doi:10.1111/pbi.70030)
Supplement: Supplementary file 1 [file PBI-23-2062-s001.docx]

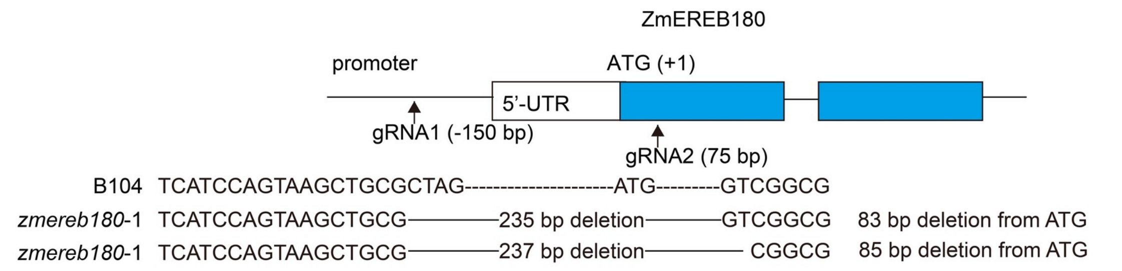
ZmEREB180
promoter
ATG (+1)
5'-UTR
gRNA1 (-150 bp)
gRNA2 (75 bp)
B104 TCATCCAGTAAGCTGCGCTAG--------------------ATG---------GTCGGCG
zmereb180-1 TCATCCAGTAAGCTGCG———235 bp deletion———GTCGGCG 83 bp deletion from ATG
zmereb180-1 TCATCCAGTAAGCTGCG———237 bp deletion——— CGGCG 85 bp deletion from ATG

zmereb180-1 TCATCCAGTAAGCTGCG————235 bp deletion————GTCGG
zmereb180-1 TCATCCAGTAAGCTGCG————237 bp deletion———— CGG
