# Supplementary material for: ZmEREB180 modulates waterlogging tolerance in maize by regulating root development and antioxidant gene expression
Source: Plant Biotechnol J. 2025 Mar 9;23(6):2062–4. doi: 10.1111/pbi.70030 (PMC12120909; doi:10.1111/pbi.70030)
Supplement: Supplementary file 2 [file PBI-23-2062-s002.docx]

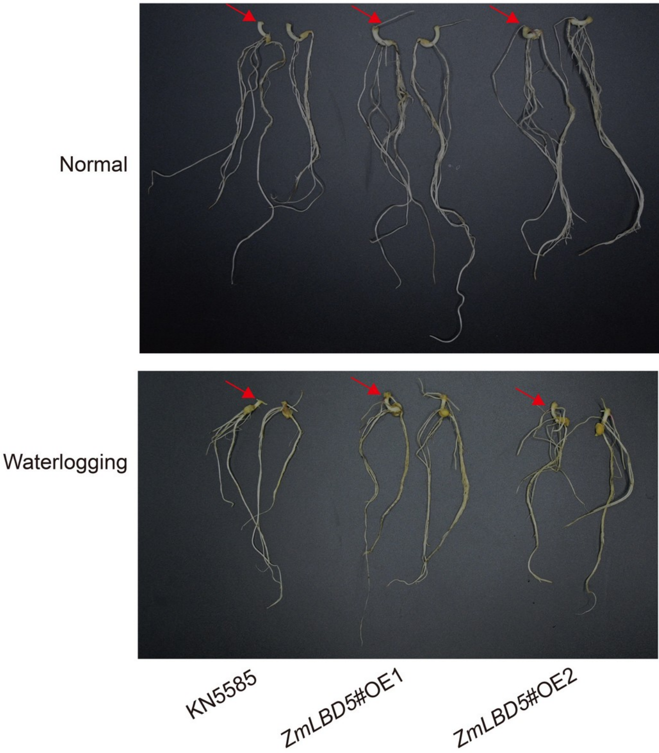
Normal
Waterlogging
KN5585
ZmLBD5#OE1
ZmLBD5#OE2

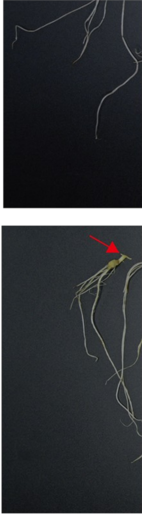
Normal
Waterlogging
KN5585
