# Supplementary material for: ZmEREB180 modulates waterlogging tolerance in maize by regulating root development and antioxidant gene expression
Source: Plant Biotechnol J. 2025 Mar 9;23(6):2062–4. doi: 10.1111/pbi.70030 (PMC12120909; doi:10.1111/pbi.70030)
Supplement: Supplementary file 3 [file PBI-23-2062-s003.docx]

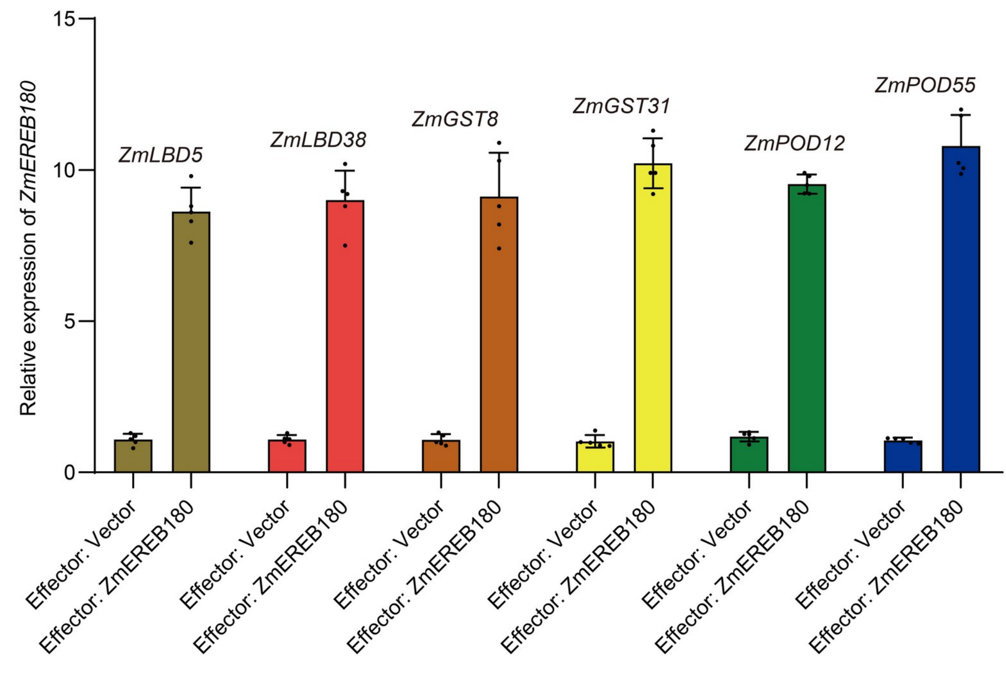

Relative expression of ZmEREB180
15
10
5
0
ZmLBD5
ZmLBD38
ZmGST8
ZmGST31
ZmPOD12
ZmPOD55
Effector: Vector
Effector: ZmEREB180
Effector: Vector
Effector: ZmEREB180
Effector: Vector
Effector: ZmEREB180
Effector: Vector
Effector: ZmEREB180
Effector: Vector
Effector: ZmEREB180
Effector: Vector
Effector: ZmEREB180

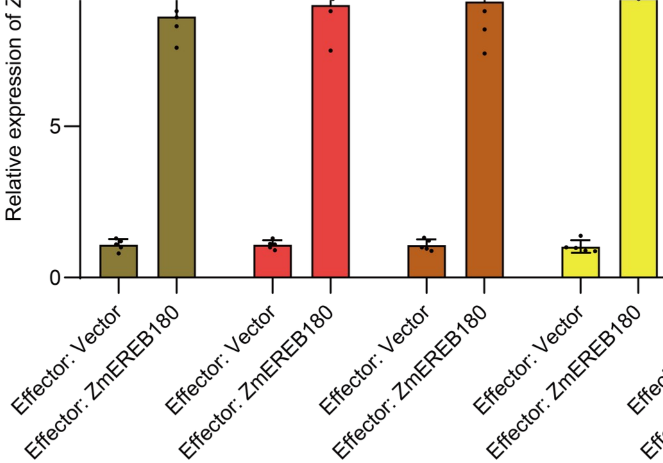

Relative expression of Z
5
0
Effector: Vector
Effector: ZmEREB180
Effector: Vector
Effector: ZmEREB180
Effector: Vector
Effector: ZmEREB180
Effector: Vector
Effector: ZmEREB180
