# Supplementary material for: ZmEREB180 modulates waterlogging tolerance in maize by regulating root development and antioxidant gene expression
Source: Plant Biotechnol J. 2025 Mar 9;23(6):2062–4. doi: 10.1111/pbi.70030 (PMC12120909; doi:10.1111/pbi.70030)
Supplement: Supplementary file 4 [file PBI-23-2062-s004.docx]

ZmLBD5 CTTAGTATAAAAAACAGCCCCGCGTCTACCCCTCCGCCGCCGCTCCATCAGCACCGTTC

GCC-box

ZmLBD38 AGGCCGCGCGGCGGCGCCTTGCACTTTTGGATTCTTCTGCCGCCTCCAGTTTCTTCG

GCC-box

ZmGST8 CCTAGCTAATAAACAAACTCACGGGGACGCGGAAGCCGCCTCCGTCTCCTAGTCCTAAC

GCC-box

ZmGST31 AGCTCTCAGAACTCGGGAAAAAAATGTCAGAGGCCGCCGTGCGTGTGATCGGCCTATGG

GCC-box

ZmPOD12 GATGGCGCCCCGCACGCTCTATGACGACGGCGGCTCTCAGCCCCCTTACGGAAGCAAGA

GCC-box

ZmPOD55 CAGCTGTGACTCTGAGCCAGGCCCATGTGCTGCCGCCCCAACGCTGCCTAGTTCCTGCG

GCC-box

ZmGST31 AGCTCTCAGAACTCGGGAAAAAAATGTCAGAGGCCGCCGTC

GCC-box

ZmPOD12 GATGGCGCCCCGCACGCTCTATGACGACGGCGGCTCTCAG

GCC-box

ZmPOD55 CAGCTGTGACTCTGAGCCAGGCCCATGTGCTGCCGCCCCA

GCC-box
