# Supplementary material for: ZmEREB180 modulates waterlogging tolerance in maize by regulating root development and antioxidant gene expression
Source: Plant Biotechnol J. 2025 Mar 9;23(6):2062–4. doi: 10.1111/pbi.70030 (PMC12120909; doi:10.1111/pbi.70030)
Supplement: Supplementary file 5 [file PBI-23-2062-s007.docx]

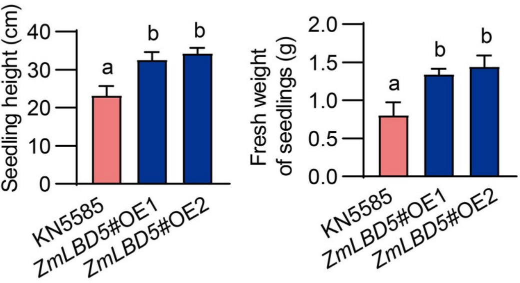
Seedling height (cm)
40
30
20
10
0
a
b
b
KN5585
ZmLBD5#OE1
ZmLBD5#OE2
Fresh weight of seedlings (g)
2.0
1.5
1.0
0.5
0.0
a
b
b
KN5585
ZmLBD5#OE1
ZmLBD5#OE2
