# Supplementary material for: ZmEREB180 modulates waterlogging tolerance in maize by regulating root development and antioxidant gene expression
Source: Plant Biotechnol J. 2025 Mar 9;23(6):2062–4. doi: 10.1111/pbi.70030 (PMC12120909; doi:10.1111/pbi.70030)
Supplement: Supplementary file 6 [file PBI-23-2062-s008.docx]

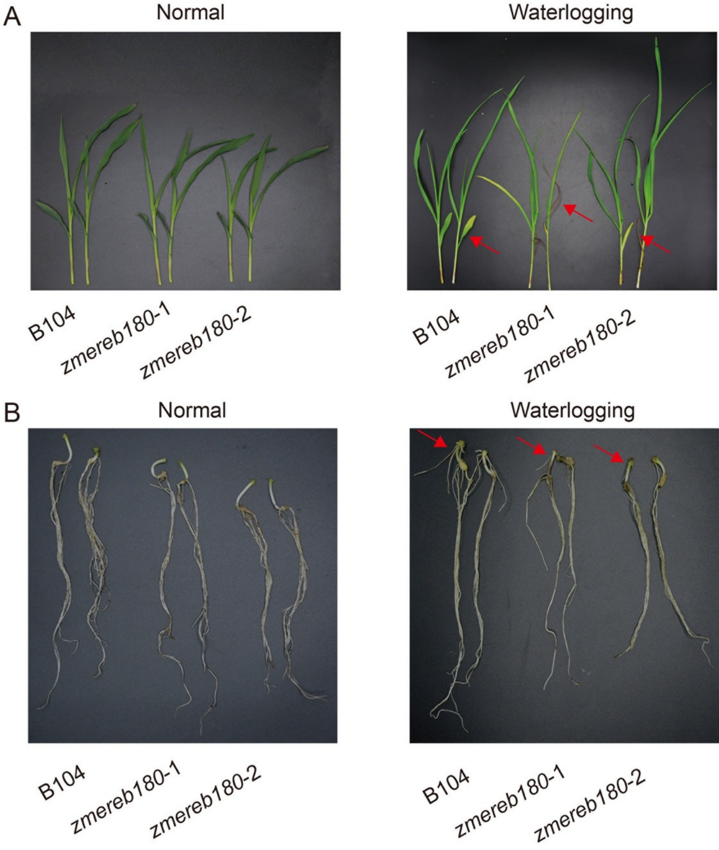
A
Normal
Waterlogging
B104
zmereb180-1
zmereb180-2
B104
zmereb180-1
zmereb180-2
B
Normal
Waterlogging
B104
zmereb180-1
zmereb180-2
B104
zmereb180-1
zmereb180-2

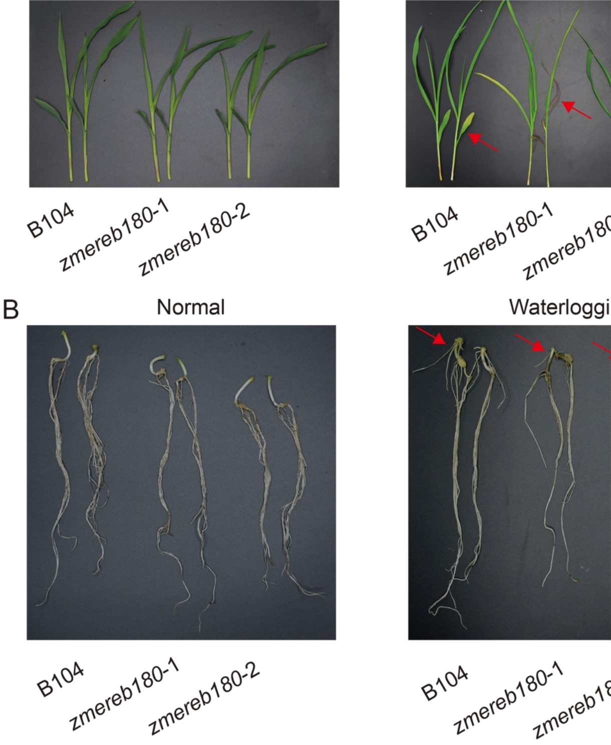

B104
zmereb180-1
zmereb180-2
B104
zmereb180-1
B
Normal
B104
zmereb180-1
zmereb180-2
B104
zmereb180-1
